# Metabolic and morphological changes of an oil accumulating trebouxiophycean alga in nitrogen-deficient conditions

**DOI:** 10.1007/s11306-012-0463-z

**Published:** 2012-09-29

**Authors:** Takuro Ito, Miho Tanaka, Haruka Shinkawa, Takashi Nakada, Yoshitaka Ano, Norihide Kurano, Tomoyoshi Soga, Masaru Tomita

**Affiliations:** 1 Institute for Advanced Biosciences, Keio University, Tsuruoka, 997-0052 Yamagata Japan; 2 Systems Biology Program, Graduate School of Media and Governance, Keio University, Fujisawa, 252-8520 Japan; 3 PRESTO, Japan Science and Technology Agency, Kawaguchi, 332-0012 Japan; 4 Research Laboratories, DENSO CORPORATION, Nisshin, 470-0111 Japan; 5 Faculty of Environment and Information Studies, Keio University, Fujisawa, 252-8520 Japan

**Keywords:** Oil-rich algae, Capillary electrophoresis, Liquid chromatography, Mass spectrometry, Metabolic profile

## Abstract

Oil-rich algae have promising potential for a next-generation biofuel feedstock. *Pseudochoricystis ellipsoidea* MBIC 11204, a novel unicellular green algal strain, accumulates a large amount of oil (lipids) in nitrogen-deficient (–N) conditions. Although the oil bodies are easily visualized by lipophilic staining in the cells, little is known about how oil bodies are metabolically synthesized. Clarifying the metabolic profiles in –N conditions is important to understand the physiological mechanisms of lipid accumulations and will be useful to optimize culture conditions efficiently produce industrial oil. Metabolome and lipidome profiles were obtained, respectively, using capillary electrophoresis- and liquid chromatography-mass spectrometry from *P. ellipsoidea* in both nitrogen-rich (+N; rapid growth) and –N conditions. Relative quantities of more than 300 metabolites were systematically compared between these two conditions. Amino acids in nitrogen assimilation and N-transporting metabolisms were decreased to 1/20 the amount, or less, in –N conditions. In lipid metabolism, the quantities of neutral lipids increased greatly in –N conditions; however, quantities of nearly all the other lipids either decreased or only changed slightly. The morphological changes in +N and –N conditions were also provided by microscopy, and we discuss their relationship to the metabolic changes. This is the first approach to understand the novel algal strain’s metabolism using a combination of wide-scale metabolome analysis and morphological analysis.

**Electronic supplementary material:**

The online version of this article (doi:10.1007/s11306-012-0463-z) contains supplementary material, which is available to authorized users.

## Introduction

Oil-rich algae are expected to be a promising source of next-generation bioenergy because microalgae have the potential to produce up to 300 times more lipids than major oil crops based on biofuel feedstock production per area (Schenk et al. 2008). Cultivating algae is also proposed as a way to absorb carbon dioxide (Wang et al. 2008). However, cultivating costs are still high and, regarding industrial use, the biological knowledge of oil accumulation mechanisms is poor (Schenk et al. 2008, Sheehan et al. 1998).

Oil-rich algae accumulate intracellularly or they secrete oil (lipids) in stress conditions, and some oil-rich algae, mainly marine algae, contain oil bodies in normal culture conditions (Shifrin and Chisholm 1981, Sheehan et al. 1998, Hu et al. 2008). Oil bodies are easily visualized with lipophilic dye (e.g. nile red (Greenspan et al. 1985, Cooksey et al. 1987)). *P. ellipsoidea* MBIC 11204 (Trebouxiophyceae, Chlorophyta), a novel unicellular green algal strain, was isolated from a hot spring in Japan, and accumulates large amounts of lipids, including diesel class hydrocarbons in –N conditions (Satoh et al. 2010). Generative growth is the other common response to –N conditions in green algae, however it is not reported in Trebouxiophyceae except *Trebouxia*. In nitrogen-rich (+N) conditions, *P. ellipsoidea* grows rapidly, but oil accumulation is limited (Satoh et al. 2010). Accumulating oil, known as lipid accumulate conditions, principally triacylglycerols (TAGs), in response to –N conditions, has been observed in numerous species or strains of various algal taxa, and also in several different culture conditions, which are other nutrient-deficient, and in chemical stimulus and physical stress (Basova 2005, Liu et al. 2008, Merzlyak et al. 2007, Roessler 1990, Shifrin and Chisholm 1981, Thompson 1996). Based on the sequence homology and some shared biochemical characteristics, researchers believe that green algae share basic lipid-synthetic pathways with higher plants; however lipid synthesis and accumulation mechanisms in stress conditions are barely characterized in algae (Hu et al. 2008, Thompson 1996).

Metabolites are products of cellular regulatory processes, and their profiles are regarded as biological systems’ responses to environmental changes (Fiehn 2002). In this decade, mass spectrometry (MS)-based metabolome analysis has become an important tool for characterizing intracellular metabolic profiles (Fiehn 2002, Soga et al. 2003, Aharoni et al. 2002, Dennis 2009). Metabolome analysis is a useful application for model organisms and for uncommon organisms because it does not require genomics or other “omics” information. Quantitative and qualitative measurements of large numbers of cellular metabolites are useful to elucidate the dynamics of biological systems; however, measuring metabolome profiles has been difficult because of the wide diversity of chemical properties, i.e., polarity (solubility), electronic charges of ions, volatility, and molecular weight (Stitt and Fernie 2003). Approximately 200,000 metabolites have been estimated to exist in the plant kingdom (Fiehn et al. 2001, Fiehn 2002). Several methods have provided wide-range metabolome analyses, including capillary electrophoresis–MS (CE–MS) (Soga et al. 2003, Soga et al. 2009, Soga et al. 2006), liquid chromatography–MS (LC–MS)(Yang et al. 2008, Ejsing et al. 2009), gas chromatography/MS (GC/MS)(Fiehn et al. 2000), nuclear magnetic resonance (Reo 2002), and Fourier transform ion cyclotron resonance-MS (Aharoni et al. 2002). In the algal study, the metabolic responses of *Chlamydomonas reinhardtii* to nutrient (nitrogen, phosphorus, sulfur, or iron) deficient, mixo- and auto-trophic conditions are currently being analyzed by GC/MS (Bölling and Fiehn 2005, Wienkoop et al. 2010).

In this study, we use CE–MS and LC–MS to focus the analysis on ionic metabolites that exist abundantly in central metabolisms, and to analyze lipids, including free fatty acids (FAs), phospholipids, glycolipids (GLs), neutral lipids (NLs), and pigments. We also performed transmission electron microscopy (TEM), fluorescent microscopy, cell density and volume analysis, and total protein quantification to understand the cell state and to consider the quantitative changes of metabolites in a cell.

## Materials and methods

### Algal material and culture conditions

*Pseudochoricystis ellipsoidea* nom. nud. MBIC 11204 (Satoh et al. 2010; international patent, PCT/JP2006/306785) was provided by DENSO CORPORATION (Aichi, Japan). This strain had been deposited as the deposition number of FERM BP-10484 in International Patent Organism Depositary (IPOD), National Institute of Advanced Industrial Science and Technology (Ibaraki, Japan). An autotrophic medium named A5, containing nitrogen as NaNO_3_ (without pH adjustment from Satoh et al. (2010); Supplementary Table. ST1), was used for growth along with a nitrogen deficient medium (A5–N); this has the same composition as A5 medium except it lacks NaNO_3_. The cultures were grown in flat-flasks (working volume: 500 ml) under continuous illumination (180 μmol m^−2^ s^−1^) and aeration (300 ml/min. with 1 % CO_2_) at 25 °C. For all examinations except TEM, the stock culture of *P. ellipsoidea* was grown in A5 for least 3 days as preculture, and then replaced into fresh A5. In exponential growth phase, the culture was sampled as “nitrogen-rich (+N) condition”. The rest of the cells was replaced into A5–N and collected as “nitrogen-deficient (–N) condition” after 3 days cultivation. For TEM, the culture was prepared without preculture.

### Cell analyses

The cell density and volume were measured by the particle analyzer, CDA-1000 (Sysmex, Japan). For total protein quantification, microalgal cells in 50 mmol/l Tris–HCl (pH 7.5) added protease inhibitor cocktail (Roche, US) were homogenized with approximately 2 g of ø 0.5 mm zirconia beads using a Micro Smash MS-100R (Tomy Seiko, Japan) at 4,000 rpm for twice of 90 s. Then added 0.25 % Triton X-100, centrifuged at 12,000×*g* for 10 min. The supernatant was used for protein determination by the brad ford assay (Bradford, 1976).

### Microscopy

Light and fluorescence microscopy was carried out using a Leica DM2500 microscope equipped with Nomarski interference optics (Leica, Germany), N3 fluorescence filter cube (excitation: 540–552 nm band-pass, suppression: 580–620 nm band-pass; Leica) for nile red fluorescence, a custom fluorescence filter cube (excitation: 340–380 nm band-pass, suppression: 425 nm long-pass; Leica) for autofluorescence and Olympus DP71 digital camera (Olympus, Japan). For observation of lipid bodies, a stock solution of nile red (33 μg/ml; Sigma-Aldrich, USA) in methyl sulfoxide containing 1 % ethanol was added to preparations to effect a 1:4 dilution. For observation of starch granules, iodine solution (0.05 mol/l; Wako, Japan) was added to preparations to effect a 1:5 dilution.

For TEM, the cells fixed for 1–4 days in 2 % glutaraldehyde in 30 mmol/l HEPES buffer, and then postfixed with osmic acid at 4 °C for 3 h. After dehydration in a graded series of ethanol (50–100 %), the samples were embedded in Epon 812 (Shell Chemical, USA) at 60 °C for 2 days. The specimens were sectioned, stained with uranyl acetate and lead citrate, and examined in a JEM-1200EX electron microscope (JEOL, Japan).

### Preparation of samples

Approximately 10^8^ cells were collected from five individual cultures of +N or –N conditions for metabolome analysis using centrifuge at 3,310×*g* for 3 min. The pellet of cells were resuspended and washed with 1 ml of Milli-Q water, and then 650 μl of methanol including 20 μmol/l each of internal standards, methionine sulfone and d-camphor-10-sulfonic acid were added.

Metabolite extraction was described previously (Soga et al. 2003), and modified for algae as below. The microalgal cells in methanol were homogenized using a Micro Smash MS-100R as described above. Genomic DNA (gDNA) was extracted from 50 μl of each suspension using FastPure DNA kit (Takara Bio, Japan) following the procedure for mammalian tissue with RNase A treatment of the product, and was quantified using Quant-iT dsDNA HS assay kit with Qubit fluorometer (Life Technologies, USA). For each sample, the measured metabolite quantity were normalized using gDNA content to obtain the amount of metabolite contained per unit cells of each sample. Then, 600 μl of chloroform including 50 μmol/l of internal standards, 1,2-dihexanoyl(d22)-*sn*-glycero-3-phosphocholine (Avanti Polar Lipids, USA), and 240 ml of Milli-Q water were added to residual suspension. The organic phase was reserved in glass vials and stored at −80 °C until analysis. The aqueous phase was also processed for analyses as described previously (Soga et al. 2003), and resolved to 25 μl Milli-Q water.

### Metabolome and lipidome analyses

The CE-TOFMS and LC–MS/MS conditions for cationic and anionic metabolite, and free sugar analyses were as described elsewhere (Hirayama et al. 2009, Soga et al. 2009, Sugimoto et al. 2010a Sugimoto et al. 2010b). The LC-TOFMS conditions for lipids are described below. For free fatty acids analysis, the samples were separated on an Ascentis Express RP-Amide column (particle size, 2.7 μm; ø 2.1 × 150 mm ID; Sigma-Aldrich, St. Louis, MO, USA) at 50 °C. The mobile phase was Milli-Q water containing 0.1 % acetic acid (A) and isopropanol (B), and the gradient condition of mobile phase started with the solvent (A/B:60/40), then linearly converting to solvent (A/B:1/99) for 40 min with holding for 5 min. Separations of the other lipids were carried out on an Ascentis Express C8 column (particle size, 2.7 μm; ø 2.1 × 150 mm ID; Sigma-Aldrich, St. Louis, MO, USA) at 50 °C. The mobile phase was Milli-Q water containing 10 mmol/l ammonium acetate (A) and isopropanol (B). The gradient started with the solvent mixture (A/B:65/45), and then linearly converted to the solvent mixture (A/B:1/99) for 40 min with holding for 5 min. For each lipid analysis, the mobile phase was pumped at a flow rate of 200 μl/min. The post time period was 15 min. Typically, 1.0 μl of sample solution was injected. An IsoPump was used to deliver the API-TOF Reference Mass Solution Kit (G1969-85001; Agilent Technologies, Santa Clara, CA, USA) for automatic recalibration. TOFMS was conducted in the negative or positive ion mode for fatty acid or other lipids analyses, respectively: the capillary voltage was set at 4 kV; nitrogen gas (350 °C) was used for electrospray ionization; the fragmenter, skimmer, and OCT RF voltage were set at 175, 60, 175 V for negative mode, and 250, 60, and 250 V for positive mode; mass spectra were acquired at the rate of 1.0 cycles/s over an *m*/*z* 100–1,200 range for negative mode and 50–1,650 for positive. In this analytical method, coefficient of variation of 5 replicate was less than 9 % for 3 selected molecular species of fatty acid, less than 5 % for 3 species of phosphatidyl ethanolamine, less than 6 % for 3 species of phosphatidyl glycerol, respectively. Raw data from CE-TOFMS and LC-TOFMS were processed with our proprietary software named MasterHands (Soga et al. 2009, Sugimoto et al. 2010b, Sugimoto et al. 2010c). For cationic and anionic metabolite, and free sugar analyses, all compounds were identified and quantified using standard compounds as described elsewhere (Hirayama et al. 2009, Soga et al. 2009, Sugimoto et al. 2010b, Sugimoto et al. 2010a). For lipid analysis, several major compounds were identified using standards (Supplementary Table. ST2), but the others were identified based on theoretical m/z values (Supplementary Table. ST3) with mass accuracy of 20 ppm and orderly shift of retention time, described by Ikeda et al. (2009). Relative quantity of lipids was calculated using the internal standard. The position of double bonds or chirality was not identified in this study. All peaks were visually confirmed.

## Results and discussion

### Morphological analyses

**Fig. 1:**
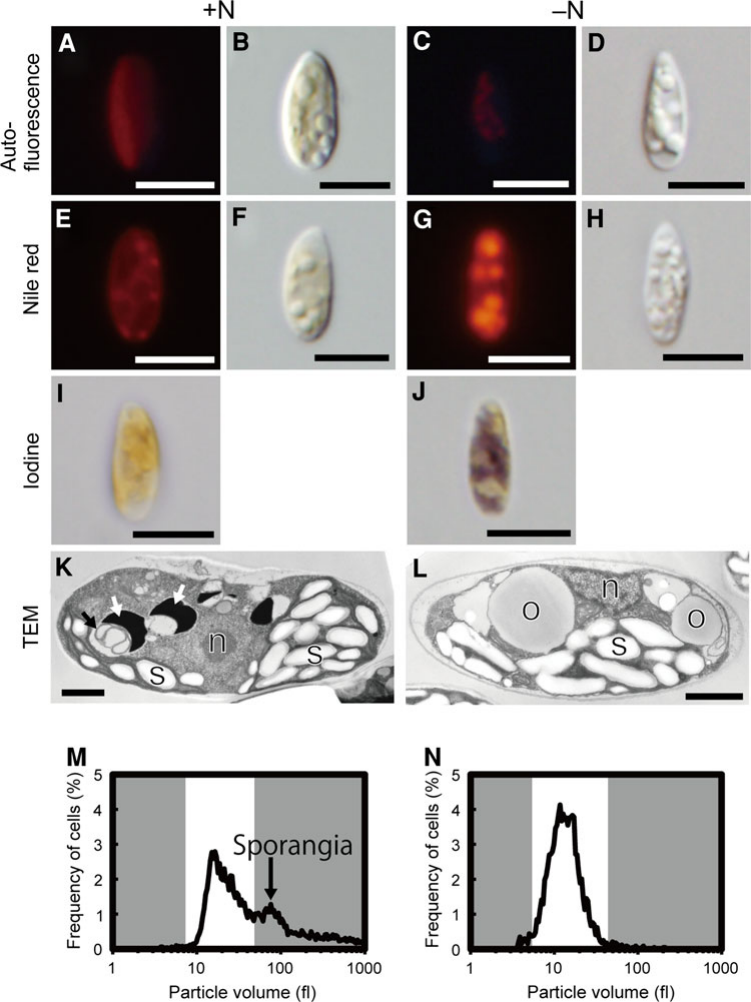
The morphological changes between nitrogen-rich and -deficient conditions. *Gray color* zone in **m**, **n** was cut off for cell volume calculation in Table 1. **a**, **c** Chlorophyll autofluorescence. **e**, **g** *Nile red* stain of oil bodies. **b**, **d**, **f** and **h** Nomarski interference images. **i**, **j** Iodine stain for starch granules. **k**, **l** Transmission electron microscopy. **m**, **n** Cell volume distributions. *Bar* length **a**–**j** 5.0 μm; **k**, **l** 1.0 μm. *o* oil body; *s* starch granule; *n* nucleus; *white arrows* large electron dense bodies (LEDBs); *black arrow* multi-membrane vesicle (MMV)

To compare cell morphology, we performed several microscopies on *P*. *ellipsoidea* in +N and –N conditions. In –N conditions, the cell width of *P*. *ellipsoidea* was shorter (usually 2.4–3.4 μm) than in +N conditions (usually 2.9–4.3 μm) in spite of having the same cell length (usually 7.2–8.6 μm) in both conditions (Fig. 1a–d). The chloroplast, observed by chlorophyll fluorescence, was smaller in –N conditions than +N conditions, and the chloroplast fluorescence was diluted in –N conditions (Fig. 1a, c). Additionally, sporangia were observed only in +N conditions, suggesting arrest of cell division in –N conditions. Cell volume distributions were quite different between +N and –N conditions. In +N conditions, the culture included a peak corresponding to sporangia (Fig. 1m), while we found only a single vegetative peak in –N conditions. The vegetative cells in –N condition were smaller than the vegetative cells in +N conditions (Fig. 1n; Table 1). Although endomembrane system was not strongly stained by nile red in +N conditions, large oil bodies were observed in –N conditions using fluorescence microscopy (Fig. 1e, g). In –N condition, many starch granules were stained by iodine, but rarely in +N conditions (Fig. 1i, j). In oil-rich green algae, increasing oil body and starch granule, and decreasing chlorophyll were general responses to –N conditions (Shifrin and Chisholm 1981, Sheehan et al. 1998, Hu et al. 2008, Msanne et al. 2012).

**Table 1:** The effects of nitrogen-deficiency for cell cultures

	+N		–N		
	mean	SD	mean	SD	–N/+N
Cell density (cells/ml)	2.2 × 10^6^	3.1 × 10^5^	3.7 × 10^6^	3.0 × 10^5^	1.9^*^
Cell volume (μm^3^)	43	1.9	17	0.25	0.26^*^
Biomass (μm^3^/ml)	9.7 × 10^7^	1.8 × 10^7^	6.1 × 10^7^	4.9 × 10^6^	0.49^*^
Total protein (ng/10^5^ cells)	27	8.1	11	1.6	0.39
pH of the culture medium	7.9	0.096	6.4	0.0	NA

*NA* not applicable

* The cell density and volume measured after replacement into A5–N (density: 1.9 × 10^6^; volume: (65) was used to calculation as +N

n = 4 for each culture condition

*Pseudochoricystis ellipsoidea* cells contained a parietal chloroplast and a nucleus in the middle of the cell (Fig. 1k, l). We observed mitochondrial profiles near the chloroplast on the inner side (Fig. 1k, l). In +N conditions, *P*. *ellipsoidea* cells sometimes contained a few small oil bodies, and we often observed several large electron dense bodies (LEDBs; Fig. 1k) in the cytoplasm. The LEDBs were not surrounded by a membrane, but were often associated with a vesicle surrounded by multiple membranes (multi-membrane vesicle: MMV; Fig. 1k). Cells grown in –N conditions did not contain LEDBs, but large oil bodies occupied a large part of the cytoplasm (Fig. 1k, l). The oil bodies were sometimes associated with the MMV, indicating LEDB and lipid body homology. Many starch granules were stored in the chloroplast in both +N and –N conditions (Fig. 1k, l). These results were different from iodine stain observations in +N conditions in which only few starch granules were observed. Because the cells for TEM analysis were cultured without pre-culturing, the cells may also include starch granules in +N conditions. In +N conditions, most of the starch granules in the chloroplast were slightly distant from each other, and several thylakoid lamellae separated them. In –N conditions, the starch granules were often fused with each other, and thylakoid lamellae were less developed in the chloroplast. In many eukaryotes including *C*. *reinhardtii* and *Arabidopsis thaliana*, autophagies are induced in stress conditions (Mizushima 2007, Pérez-Pérez et al. 2010, Wada et al. 2009), and the chloroplast reduction of *P. ellipsoidea* in –N conditions might be explained by autophagy.

### The culture in nitrogen-rich and -deficient conditions

Cell density, cell volume, biomass, total protein abundance, and culture pH in both +N and –N conditions were examined for basic cell information. Cell density of 3-day cultures was 1.9 times higher (3.7 × 10^6^ cells/ml) than 0-day cultures (1.9 × 10^6^ cells/ml) in –N conditions (Table 1). The cell density was increased but not decreased least 5 days after treatment (data not shown,) therefore cells should be survive in this 3-day treatment. Biomass volume par unit culture was decrease to a half while cell density was increased, because cell volume was decreased to 1/4 (Table 1). The average cell volume was determinably shifted to a small size in –N conditions because the cells had divided without vegetative growth (Fig. 1m, n). Total protein abundance decreased to 1/2.6 (Table 1), because nitrogen nutrient, NaNO_3_ in this medium, is requested to de novo protein synthesis.

The pH of the algal culture medium was 7.9 in +N conditions, and 6.4 in –N conditions, while the pH of flesh media was 6.8. Cells took a sufficient amount of carbon dioxide in +N conditions, but cells may have considerably reduced carbon dioxide uptake in –N conditions and the pH of the culture medium decreased to 6.4. Otherwise, cells may have secreted some acidic compounds in –N conditions. In the future, metabolome analysis of culture media may elucidate the secreted metabolites.

### Metabolome and lipidome analyses

**Fig. 2:**
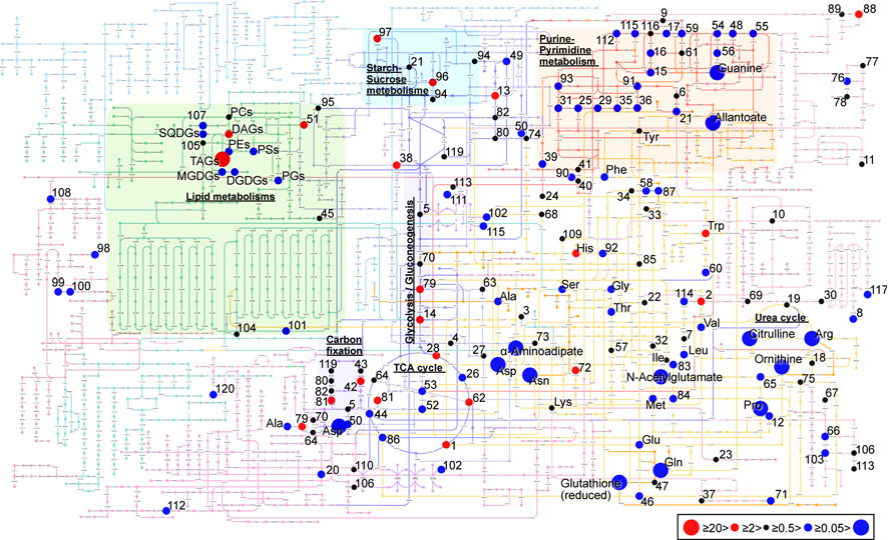
The quantitative changes of detected metabolites mapped on the KEGG Atlas. The names of the metabolites are shown in Supplementary Table. ST2. Metabolites not included in the KEGG Atlas (185 metabolites) are not shown. Large *red dot* –N/+ N ≥ 20; Small *red dot* 20 > –N/+ N ≥ 2; *Black dot* 2 > –N/+ N ≥ 0.5; Small *blue dot* 0.5 > –N/+ N ≥ 0.05; Large *blue dot* –N/+ N < 0.05

To determine the metabolic effect of –N conditions in *P. ellipsoidea*, metabolome and lipidome analyses were performed in +N and –N conditions using CE- and LC-TOFMS, and LC–MS/MS. The supplementary material (Supplementary Table. ST2) shows 329 metabolites derived from data processing in this study; 45 % (149) of the metabolites decreased in –N conditions at *p* < 0.05 based on the Mann–Whitney *U*-test, while only 11 % (36) of metabolites increased. The quantity of metabolites in extracts was normalized by genomic DNA content. Then, we calculated an average, SD and a *U*-test *p* value from five different cultures of +N or –N conditions. Using the KEGG Atlas (Okuda et al. 2008), a global metabolism map, and the Pathway Projector (Kono et al. 2009), 67 % (221 metabolites) of detected metabolites were visualized (Fig. 2). The others were not described on the KEGG Atlas.

**Fig. 3:**
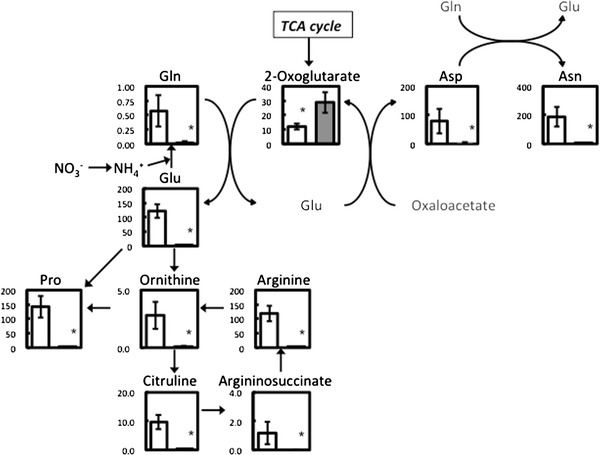
The metabolic profile of nitrogen assimilation and N-transporting metabolism in nitrogen-rich and -deficient conditions. *Columns* the quantity (μmol/l) in extracts normalized by genomic DNA content (μg). *Bars* SD; *white bars* +N condition; *Gray bars* –N condition. *p < 0.05 of the Mann–Whitney *U*-test

**Fig. 4:**
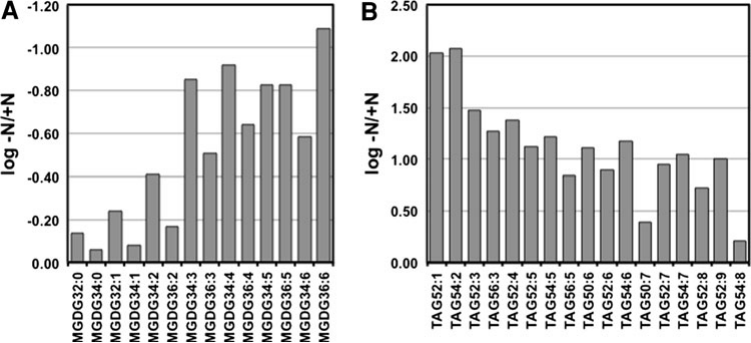
The –N/+N ratio of monogalactosyldiacylglycerols (MGDGs; **a**) and triacylglycerols (TAGs; **b**). The numbers following the abbreviations of the lipid class indicates the total carbon chain length and total degree of unsaturation

In –N conditions, 16 metabolites were greatly decreased to 1/20 or less compared to +N conditions (Fig. 2; Supplementary Table. ST2). Some of these metabolites (Arg, Gln, Asn, Citrulline, Pro, Ornithine, and Asp) were involved in nitrogen assimilation and the N-transporting metabolism. Drastic reductions of those that have amino groups were attributed to quit the de novo synthesis of free amino acid because of the nitrogen nutrition deficiency (Fig. 3). In contrast, 2-oxoglutarate, isocitrate and citrate in the TCA cycle, precursors to Glu, significantly increased 2.4, 4.7 and 15 times, respectively (Figs. 2, 3; Supplementary Table. ST2). Intermediate of isocitrate, cis-aconitate, was decreased to a half, but it has no significant difference on the Mann–Whitney *U*-test (Fig. 2; Supplementary Table. ST2). In the downstream metabolism of 2-oxoglutarate in the TCA cycle, succinate and fumarate were significantly decreased 0.37 and 0.27 times, respectively. Therefore, the TCA cycle may be inhibited in –N conditions. Although most of the α-amino acids and protein abundance decreased in –N conditions (Fig. 2; Table 1), His and Trp significantly increased 2.6 and 6.8 times, respectively (Fig. 2); it might be explained by the reduction of protein synthesis in –N conditions. In *P. ellipsoidea*, His and Trp might be use to protein synthesis mainly, while the other amino acid might be reusable for other metabolic pathway. Other than α-amino acids, allantoate and guanine categorized into purine-pyrimidine metabolism also greatly decreased in –N conditions (Fig. 2). Most of the detected metabolites containing nitrogen atom(s) decreased in –N conditions (mainly right half of Fig. 2). Additionally, almost half (149) of the detected metabolites decreased by 1/2–1/20 in –N conditions (Supplementary Table. ST2). On the other hand, 34 metabolites increased by 2–20 times, but only four metabolites increased over 20 times in –N conditions (Supplementary Table. ST2). All four of those metabolites were molecular species of TAGs that are major components of algal oil bodies, in general. Low degree of unsaturation (1 and 2) in TAGs interestingly showed high increasing ratio (108 and 85 times, respectively) in –N conditions, while high degree (3 or more) showed relatively low increasing ratio (Fig. 4b; Supplementary Table. ST2). Non- or one-unsaturated fatty acid may be synthesized de novo in –N conditions and accumulated as TAGs in oil bodies. In starch-sucrose metabolism and glycolysis/gluconeogenesis, four out of eight metabolites increased in –N conditions (Fig. 2). This may relate to the accumulation of starch granules in –N conditions (Fig. 1i, j). Ribose 1,5-bisphosphate and fructose 1,6-diphosphate in carbon fixation (Calvin) cycle and/or pentose phosphate pathway were increased 15 and 11 times, respectively (Supplementary Table. ST2). The other 127 metabolites changed slightly or were not detected in +N or –N conditions (Supplementary Table. ST2).

Fatty acids were composed mainly of 14–18 long carbon chains without a double bond or of one double bond, as some general algae case (Harwood, 1998, Roessler, 1990), and *P. ellipsoidea* additionally contained FA22:1 (Supplementary Table. ST2). This FA composition was not significantly changed between –N and +N conditions. Because chloroplasts typically contain large amounts of pigments (Chlorophyll a and b, and beta-carotene) and GLs (MGDGs, DGDGs, and SQDGs) as thylakoid and envelope membranes (Thompson 1996), the decreasing amount of these pigments and lipids correlates to the morphological reduction of chloroplasts (Fig. 1a–d, k, l). MGDGs are usually major glycerolipids in green algal cell, and contain polyunsaturated acyl chain (Thompson 1996). In *P. ellipsoidea*, highly unsaturated MGDGs were detected, and greatly decreased compare to low unsaturated MGDGs (Fig. 4a; Supplementary Table. ST2). According to Satoh et al. (2010), acyl chain of 16:1, 16:2, 16:3, 18:2 and 18:3 in total lipid are decreased, and 16:0, 18:0 and 18:1 are increased in –N conditions in *P. ellipsoidea*. The similar change of acyl chain composition is shown in *Chlorella vulgaris*, *Scenedesmus obliquus* and *C. reinhardtii* in –N conditions (Piorreck et al. 1984; Siaut et al. 2011). In this study, highly (3 or more) unsaturated GLs were decreased, and NLs including low (less than 2) unsaturated TAGs were increased in –N conditions (Fig. 4; Supplementary Table. ST2). The highly unsaturated acyl chains in GLs were may recycled to highly unsaturated TAG. However, absolute quantification of each GL in mol-level is required to discuss this. While DAGs increased 1.5–5.6 times, TAGs increased 5.2–118 times in –N conditions (Supplementary Table. ST2). The large amount of NLs (DAGs and TAGs) that accumulated corresponds to the appearance of large sized oil bodies (Fig. 1e–h, k, l).

Bölling and Fiehn (2005) reported the metabolome profiles of *C. reinhardtii*, a model organism of chlorophycean algae, in +N and –N conditions using GC-TOFMS. Some of the results in this study differ from their results. Although citrate, fructose 1,6-diphosphate, 6-phosphogluconate and pyruvate decreased in –N conditions in this study, and Thr increased (Supplementary Table. ST2), these metabolites changed in an opposite way in *C. reinhardtii* (Bölling and Fiehn 2005). Asn, Asp, citramalate, citrulline, Gln, 3-hydroxybutyrate and Pro showed large differences in the ratios of the change between *P. ellipsoidea* and *C. reinhardtii,* even though these metabolites decreased in both species (Supplementary Table. ST2; Bölling and Fiehn 2005). One of the possible causes of these differences in metabolome profiles is the differing culture condition. *P. ellipsoidea* was photoautotrophically cultured in an A5 medium, which is a completely inorganic medium, and *C. reinhardtii* was mixotrophically cultured in a Tris–acetate-phosphate medium (Gorman and Levine 1965), which contains acetic acid, an organic nutrient (Bölling and Fiehn 2005). Otherwise the metabolic differences between the two species may be reflected in the metabolome profiles.

## Conclusion

The morphological characteristics and metabolome profiles of the oil-rich alga *P. ellipsoidea* exposed to +N and –N conditions were analyzed and compared to determine how lipids synthesize and the mechanisms in which they accumulate in –N conditions. In –N conditions, *P*. *ellipsoidea* reduced in cell size, because A5–N does not include nitrogen source for de novo synthesis of amino acids, and the cell divisions continued in this oligotrophic environment. However, characteristic increasing of His and Trp was detected, while most of N-containing metabolites were decreased. As storage products, oil bodies and starch granules were observed in –N conditions, and NLs and some metabolites on starch-sucrose metabolism and glycolysis/gluconeogenesis increased in –N conditions. In contrast to increasing NLs, other lipids were decreased or only changed slightly. Decreasing pigments and GLs can be explained by a reduction of chloroplasts. Changing PLs content might be reflects reducing endomembrane system affected by low metabolic activity. The highly unsaturated GLs, mainly MGDGs, were decreased, and NLs, mainly low unsaturated TAGs, were increased in –N conditions. Few hypotheses were proposed in this study, however the analyses were snapshots of metabolite quantities. To analyze these hypothetic metabolisms, advanced studies (e.g. metabolic flux analysis, turnover analysis, pulse-chase experiment and so on) are required. These results indicate that the combination of metabolome analyses and cell morphological analyses was more effective at starting a physiological study for novel species and strains.

## Electronic supplementary material

Below is the link to the electronic supplementary material.

Supplementary material 1 (XLS 85 kb)

Supplementary material 2 (XLS 56 kb)

Supplementary material 3 (XLS 37 kb)
